# Supplementary material for: Reconstitution of multistep recruitment of ULK1 to membranes in autophagy
Source: bioRxiv. 2025 Nov 9:2025.11.07.687251. Preprint. [Version 1] doi: 10.1101/2025.11.07.687251 (PMC12637519; doi:10.1101/2025.11.07.687251)
Supplement: 1 [file NIHPP2025.11.07.687251V1-supplement-1.pdf]

bioRxiv preprint doi: https://doi.org/10.1101/2025.11.07.687251; this version posted November 9, 2025. The copyright holder for this preprint (which was not certified by peer review) is the author/funder, who has granted bioRxiv a license to display the preprint in perpetuity. It is made available under aCC-BY 4.0 International license.

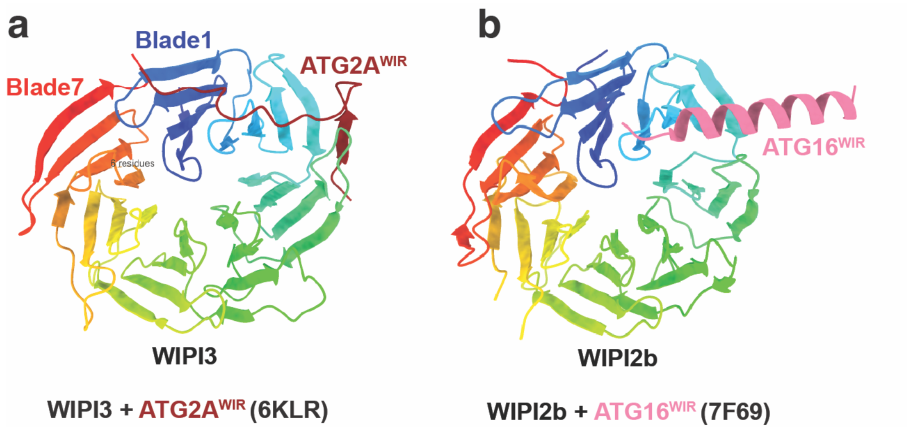

Figure S1. Comparison of the predicted binding between WIPI effectors.

(a) Crystal structure (PDB: 6KLR) of the ATG2A WIR motif bound to WIPI3 in the pockets between blades1 and 2, and blades2 and 3. **(c)** Crystal structure (PDB: 7F69) of ATG16 W2IR motif bound to WIPI2 at the cognate blade2 and 3 pocket of WIPI2.

bioRxiv preprint doi: https://doi.org/10.1101/2025.11.07.687251; this version posted November 9, 2025. The copyright holder for this preprint (which was not certified by peer review) is the author/funder, who has granted bioRxiv a license to display the preprint in perpetuity. It is made available under aCC-BY 4.0 International license.

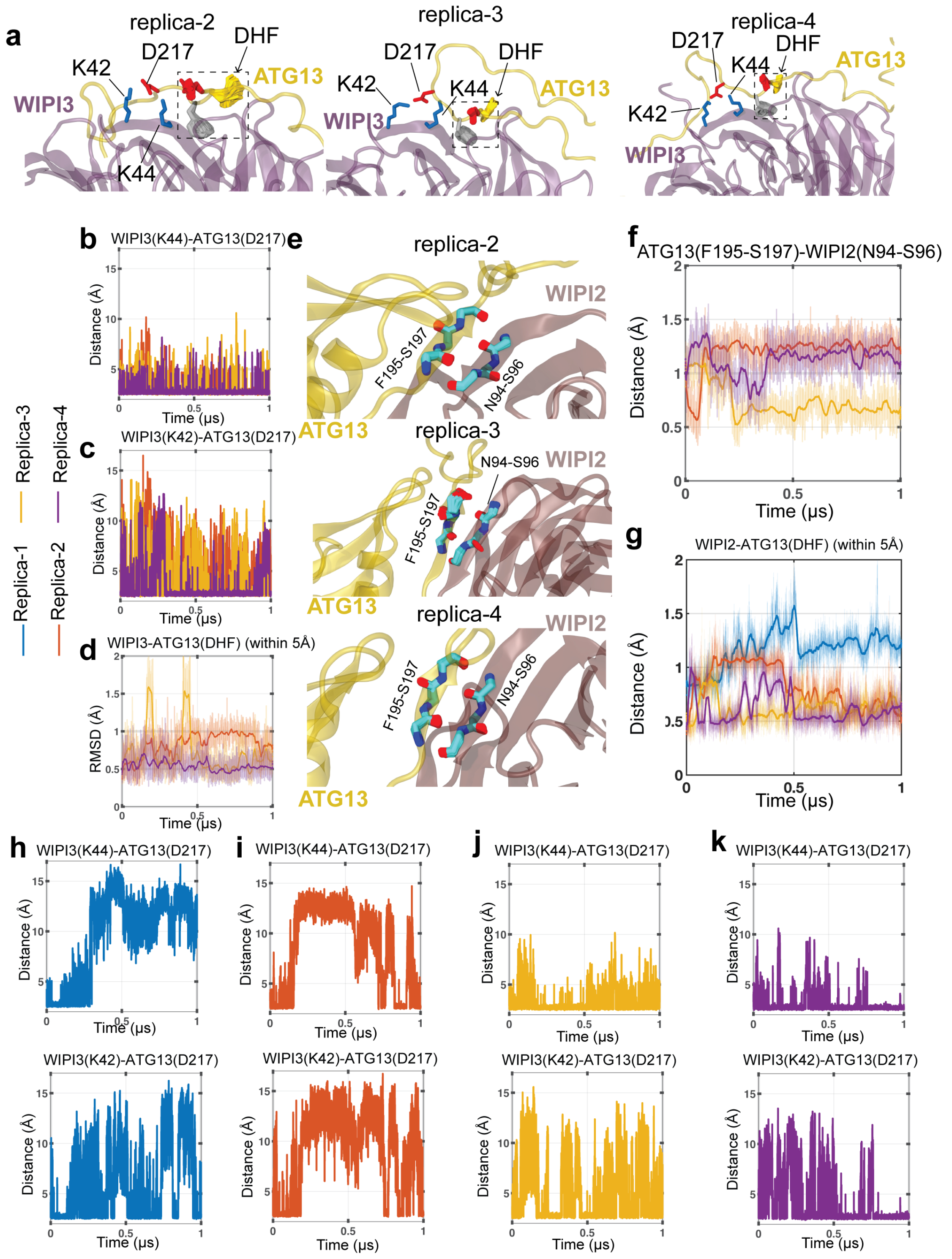

Figure S2. Dynamics of membrane-bound ATG13–ATG101–WIPI3 and ATG13–ATG101–WIPI3–WIPI2 complexes across multiple replica simulations.

bioRxiv preprint doi: https://doi.org/10.1101/2025.11.07.687251; this version posted November 9, 2025. The copyright holder for this preprint (which was not certified by peer review) is the author/funder, who has granted bioRxiv a license to display the preprint in perpetuity. It is made available under aCC-BY 4.0 International license.

Dynamics of membrane-bound ATG13–ATG101–WIPI3 and ATG13–ATG101–WIPI3–WIPI2 complexes across multiple replica simulations. **(a)** Zoomed- in view of the ATG13–WIPI3 interface (as shown in Fig. 3b for replica 1) in replicas 2, 3, and 4 of the ATG13–ATG101–WIPI3 simulation. **(b, c)** Time evolution of the minimum distance between the OD1/OD2 atoms of ATG13 D217 and the NZ atom of WIPI3 K44 (b) or K42 (c) in replicas 2–4 (replica 1 shown in Fig. 3c, 3d). **(d)** Backbone RMSD of the ATG13 DHF motif and WIPI3 residues within 5 Å of this region for replicas 2–4 (replica 1 shown in Fig. 3e). **(e)** Zoomed-in views of the ATG13–WIPI2 interface in replicas 2, 3, and 4 of the ATG13–ATG101–WIPI3–WIPI2 simulation. **(f)** Backbone RMSD of the residues shown in (f) as a function of simulation time across different replicas (replica 1 shown in Fig. 3i). **(g)** Backbone RMSD evolution of the ATG13–WIPI3 interface (as defined in D for ATG13-ATG101- WIPI3) in different replicas of the ATG13–ATG101–WIPI3–WIPI2 simulation. **(h-k)** Time evolution of the minimum distance between the OD1/OD2 atoms of ATG13 D217 and the NZ atoms of WIPI3 K44 and K42 in replicas 1 (h), 2 (i), 3 (j), and 4 (k) of the ATG13–ATG101–WIPI3–WIPI2 simulation.

bioRxiv preprint doi: https://doi.org/10.1101/2025.11.07.687251; this version posted November 9, 2025. The copyright holder for this preprint (which was not certified by peer review) is the author/funder, who has granted bioRxiv a license to display the preprint in perpetuity. It is made available under aCC-BY 4.0 International license.

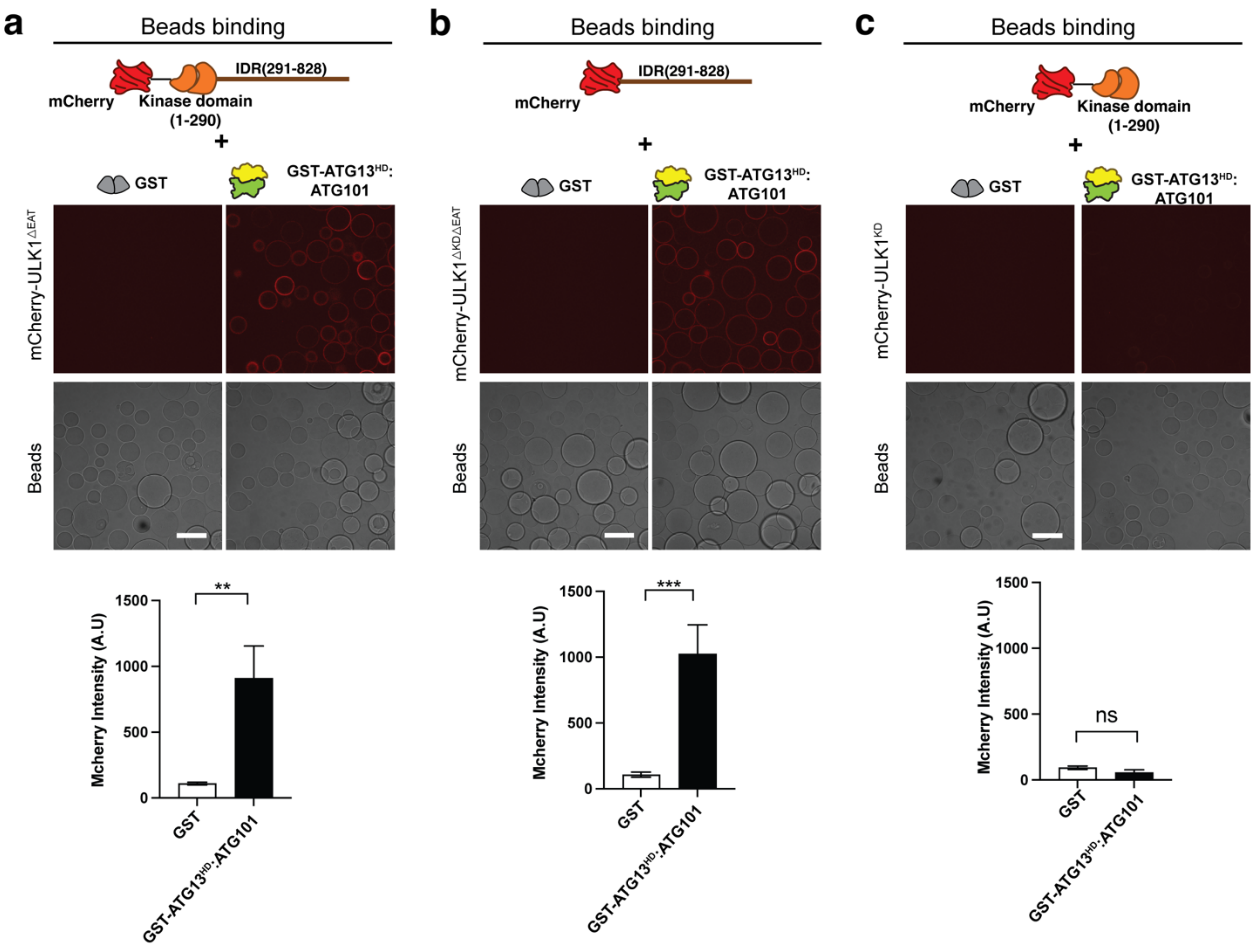

Figure S3. The IDR of ULK1 binds to ATG13(HORMA)/ATG101 directly

(a) ULK1 constructs lacking the EAT domain can bind to GST-ATG13^{HD}:ATG101, the bar graph in below showed the quantification of intensity of mcherry channel per bead; **(b)** ULK1-IDR maintain the ability that bind to GST-ATG13^{HD}:ATG101, the quantification showed in below. **(c)** ULK1^{KD} lost its binding to GST-ATG13^{HD}:ATG101. Scale bar is 100 µm. Mean ± SEM was shown. * **P<0.01, ***P<0.001, ns, non-significance.

bioRxiv preprint doi: https://doi.org/10.1101/2025.11.07.687251; this version posted November 9, 2025. The copyright holder for this preprint (which was not certified by peer review) is the author/funder, who has granted bioRxiv a license to display the preprint in perpetuity. It is made available under aCC-BY 4.0 International license.

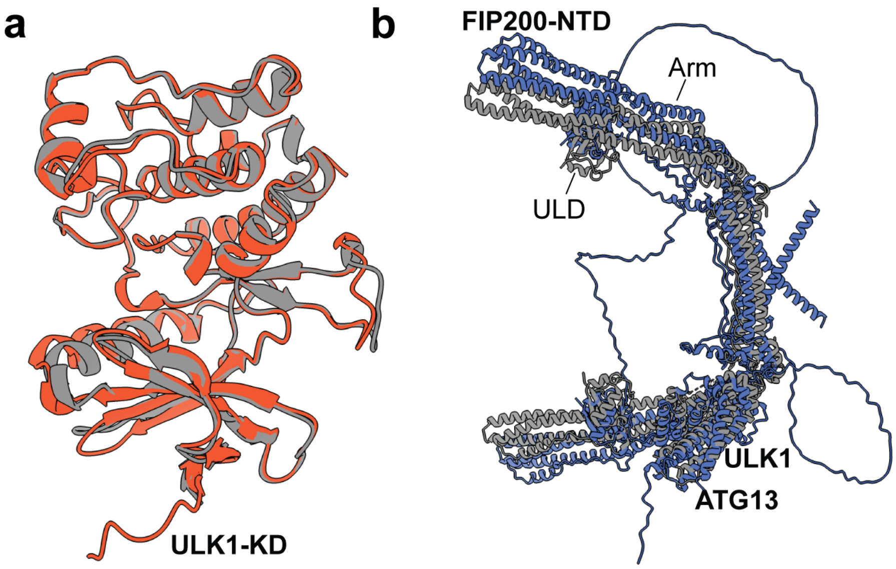

Figure S4: AlphaFold2 models used of ULK1-KD and ULK1C core.

(a) Overlay of ULK1-KD AlphaFold2 prediction (orange) with PDB ID 4WNO (grey). C_a RMSD for experimentally resolved residues is 1.3 Å. **(b)** Overlay of ULK1C core AlphaFold2 prediction (blue) with PDB ID 8SOI (grey). C_a RMSD for experimentally resolved residues is 9.6 Å.
